# Caregiving Networks and Future Care Planning of Older LGBTQ+ Adults

**DOI:** 10.1093/geroni/igaf122.3726

**Published:** 2025-12-31

**Authors:** Barbara Castro Sanchez, Sophia Tsuker, Natasha Nemmers, Amanda Leggett

**Affiliations:** Wayne State University, Detroit, Michigan, United States; Wayne State University, Detroit, Michigan, United States; Wayne State University, Detroit, Michigan, United States; Wayne State University, Detroit, Michigan, United States

## Abstract

LGBTQ+ older adults experience significant health disparities, including increased risk for chronic conditions that may necessitate formal or informal care. Many rely on chosen families rather than biological kin for caregiving, which can lead to challenges in recognition by healthcare and legal systems. Past literature has examined LGBTQ+ adults’ social networks and primary caregivers; however, little is known about how these networks translate into available or actual caregiving arrangements in later life. In-depth qualitative interviews with a focus on social networks and potential or actual caregiving networks were conducted with 12 community-dwelling LGBTQ+ older adults. Participants were aged 55 to 80 with 50% female- and 50% male-identifying. A thematic analysis identified three themes shaping caregiving networks and planning. (1) Partnership status: single participants anticipated relying on friends and chosen families, while partnered participants were more likely to list family members as potential caregivers. Single participants expressed more concerns over future care planning, listing more deliberate, active future care plans, compared to partnered individuals who reported expectations to rely on their partner. (2) Age and caregiving experience: older participants had more caregiving experience, either as providers or recipients, and more established caregiving networks. (3) The sociopolitical climate: participants expressed significant concern about the broader political climate and its long-term implications often more so than experiencing direct discrimination from individuals or healthcare providers. As aging researchers and practitioners prepare for the needs of an increasingly diverse older population, attention to LGBTQ+ caregiving dynamic and care planning is critical.

